# Effect of concurrent joule heat and charge trapping on RESET for NbAlO fabricated by atomic layer deposition

**DOI:** 10.1186/1556-276X-8-91

**Published:** 2013-02-19

**Authors:** Peng Zhou, Li Ye, Qing Qing Sun, Peng Fei Wang, An Quan Jiang, Shi Jin Ding, David Wei Zhang

**Affiliations:** 1 ASIC & System State Key Lab, School of Microelectronics, Fudan University, Shanghai, 200433, China

**Keywords:** RESET process, RRAM, Joule heat, charge trapping

## Abstract

The RESET process of NbAlO-based resistive switching memory devices fabricated by atomic layer deposition is investigated at low temperatures from 80 to 200 K. We observed that the conduction mechanism of high resistance state changed from hopping conduction to Frenkel-Poole conduction with elevated temperature. It is found that the conductive filament rupture in RRAM RESET process can be attributed not only to the Joule heat generated by internal current flow through a filament but also to the charge trap/detrapping effect. The RESET current decreases upon heating. Meanwhile, the energy consumption also decreases exponentially. This phenomenon indicates the temperature-related charge trap/detrapping process which contributes to the RESET besides direct Joule heat.

## Background

NbAlO-based resistive random-access memory (RRAM) with highly uniform bipolar resistive switching behavior has been proposed for the embedded application with multi-level storage capability and excellent reliability [1]. Generally, based on the well-accepted conductive filament hypothesis to explain the memory functional performance, several nanometer-sized filaments are indeed found in the so-called forming process. However, the conductive filament model could not clarify the origin of energy. Recently, the random circuit breaker network model [2,3] and conical shape filament model [4,5] are differently developed to emphasize joule heat contribution on breaker and thermochemical-type resistance switching, respectively. The long switching time and large power consumption of RESET (transition from a low resistance state (LRS) to a high resistance state (HRS)) process need improvements [6]. Therefore, it is important to understand the joule heat generation in resistive switching RESET behavior for the fundamental understanding. A general thermal chemical reaction (TCR) model for the RESET process has been studied by calculating the filament temperature [7]. However, we found that only the TCR itself could not explain the whole RESET process, especially for the RESET behaviors at different temperatures. In this work, we investigated the RESET process of NbAlO-based resistive switching memory device in detail at low temperatures and clarified the involved charge trapping effect.

## Methods

A NbAlO film (10 nm) was fabricated on a Pt/SiO_2_/Si substrate via atomic layer deposition (ALD) at 300°C using Al(CH_3_)_3_ and Nb(OC_2_H_5_)_5_ as the precursor and H_2_O as the oxygen source. After deposition, the sample was post-annealed in O_2_ ambient at 400°C for 10 min. The TiN top electrodes with the diameter of 100 μm were fabricated by reactive magnetron sputtering. Chemical bonding state and the microstructure of the NbAlO layer was measured through X-ray photoelectron spectroscopy (XPS) and transmission electron microscopy (TEM), respectively. The compositions of NbAlO were 1:2:5.5, as confirmed through Rutherford backscattering methods. The samples were placed on a cryogenic Lakeshore probe station (Lake Shore Cryotronics, Inc., Westerville, USA) and cooled with nitrogen liquid. The electrical characteristics were measured at increasing temperatures from 80 to 200 K in an interval of 10 K using a Keithley 4200-SCS semiconductor parameter analyzer (Keithley Instruments Inc., Ohio, USA) with the voltage applied on top electrode of TiN while the bottom Pt electrode was grounded. Because of the overshoot phenomenon with a small current compliance [8], 5 mA was chosen as the current compliance to protect the samples from electrical breakdown during the SET (transition from HRS to LRS) process.

## Results and discussion

**Figure 1:**
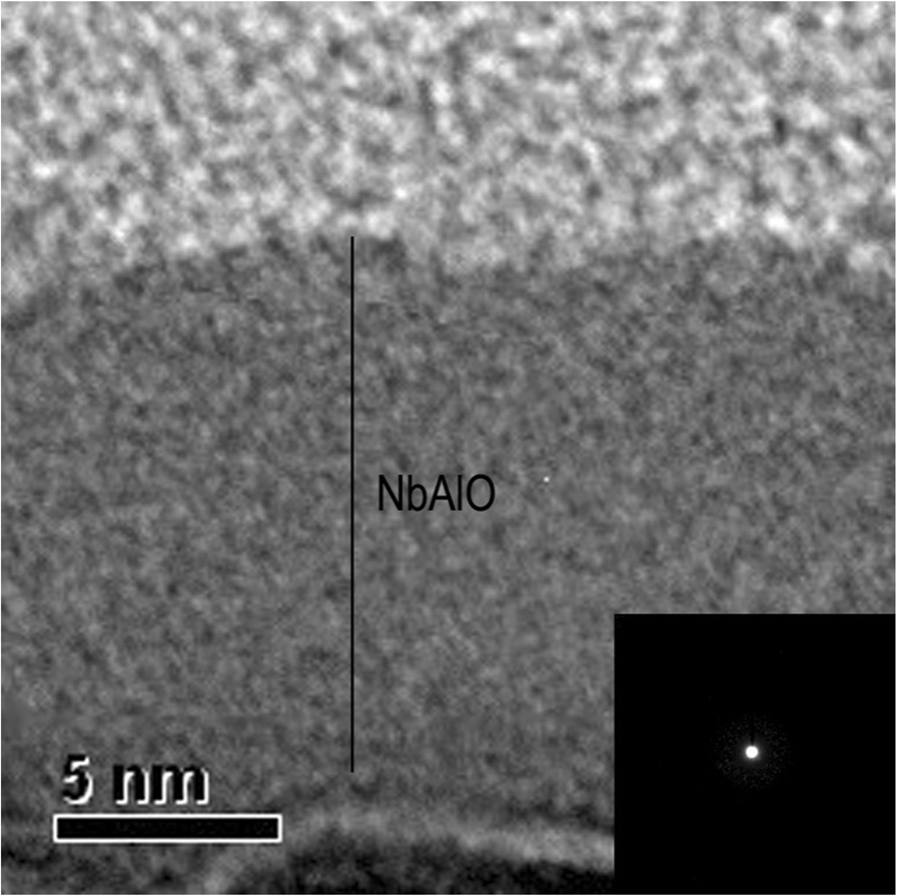
The cross-sectional TEM image of NbAlO film.

**Figure 2:**
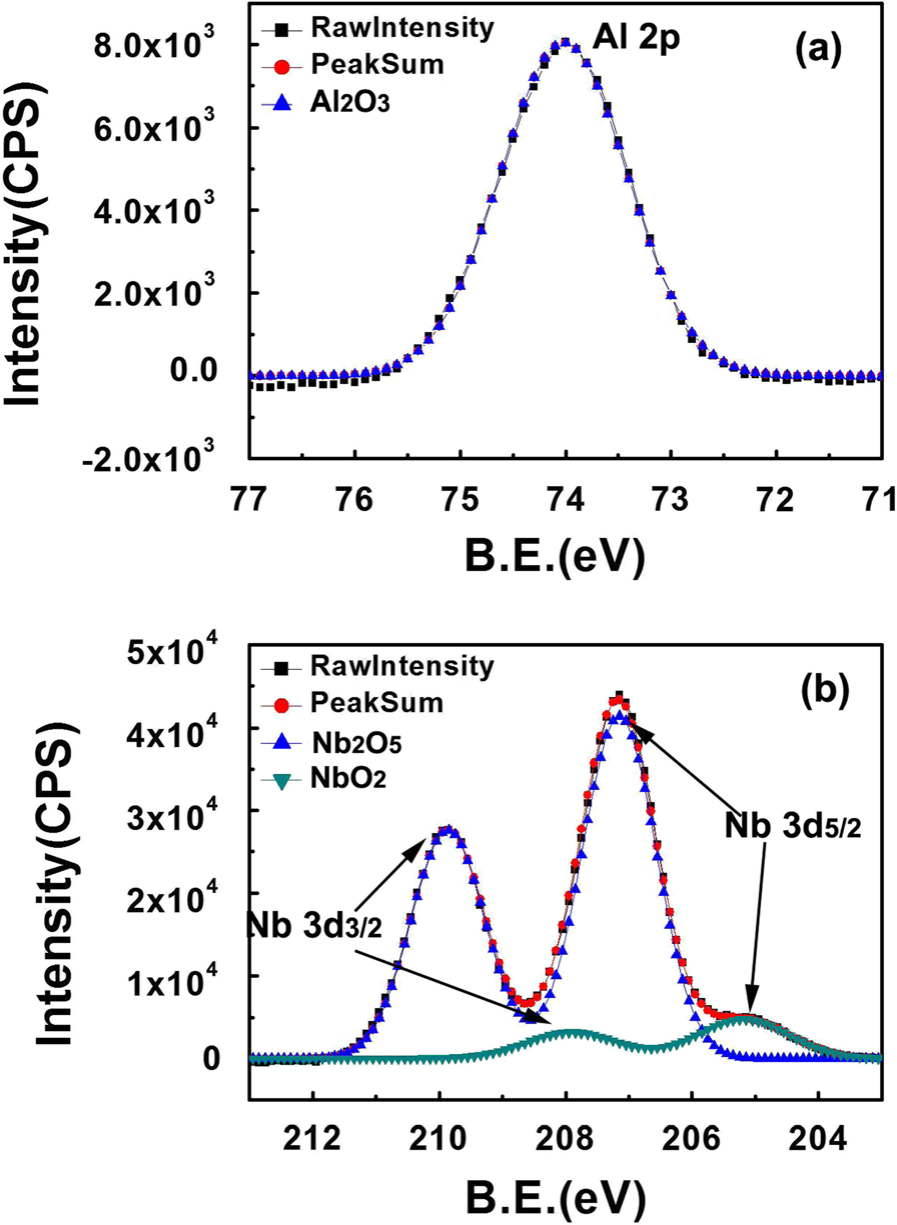
**The XPS spectra of NbAlO film chemical composition.** (**a**) The Al 2*p* peak shows the Al_2_O_3_ and (**b**) the Nb 3*d*_3/2_ and 3*d*_5/2_ peaks show the Nb_2_O_5_ and NbO_2_, respectively. The B.E. means binding energy in *x*-axis.

**Figure 3:**
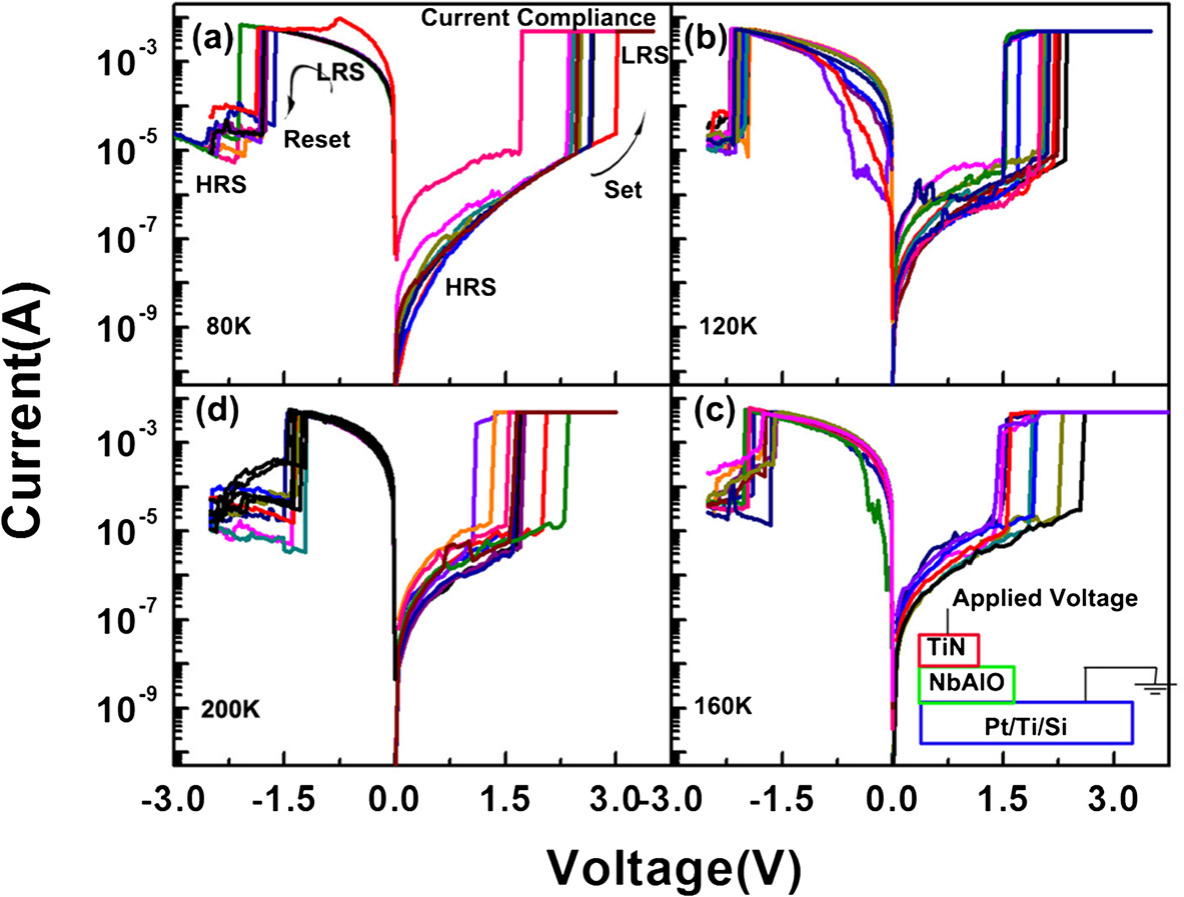
**The typical resistive switching current–voltage curve of NbAlO-based RRAM device at different environmental temperatures.** (**a**) 80, (**b**) 120, (**c**) 160, and (**d**) 200 K. The inset in (**c**) shows the schematic diagram of measured device structure and configuration. The *I*-*V* curve in different color indicates different resistive switching cycles.

From the cross-sectional TEM image of the NbAlO film, as presented in Figure 1, it was found that the NbAlO film has an amorphous structure, as further confirmed from the electron diffraction pattern in the inset of Figure 1. Most oxides grown by ALD technique at 300°C are normally amorphous. In this study, the process temperature is 300°C, while the crystallized temperatures of Nb_2_O_5_ and Al_2_O_3_ are both above 400°C. The chemical compositions of NbAlO films were shown in Figure 2. Figure 2a presents the Al 2*p* spectrum of the film. The peak position is found to be at the 74.4 eV, which indicates that Al tends to be oxidized. The Nb 3*d* spectra can be divided into two edge splits: Nb 3*d*_3/2_ and Nb 3*d*_5/2_. The Nb 3*d*_3/2_ and *3*d_5/2_ peaks are located at 210.2 eV for Nb_2_O_5_[9] and 207.5 eV for NbO_2_[10]. Figure 3 shows the typical bipolar resistive switching characteristics of NbAlO films at temperatures 80 to 200 K. By sweeping the positive voltage above a certain value (1.5 to 3 V), an abrupt current increase occurs, indicating the film in LRS. It means that the so-called SET process occurs. There is no obvious difference after more than 1,000 cycles for the current–voltage switching behavior from 80 to 200 K, as shown in Figure 3. It suggests that the conductive filaments statistically formed in the SET process have the same density, diameter, and current conduction. Hence, the difference in RESET current and energy consumption cannot be as ascribed to the random variation of uncertain conductive filament formation. In other words, the effect of SET process on the RESET difference can be safely excluded. Meanwhile, current–voltage curves after the RESET process in many cycles also keep the same route, indicative of the high repeatability of RESET characteristics of the NbAlO film, which facilitates our quantitative calculation and simulation of the process in the following research. To clarify this difference and to understand the mechanism of the RESET process, we consider the RESET from an energy point of view combined with joule heat-induced interface thermal reaction [7] and charge trap/detrapping effect [11-14]. 

**Figure 4:**
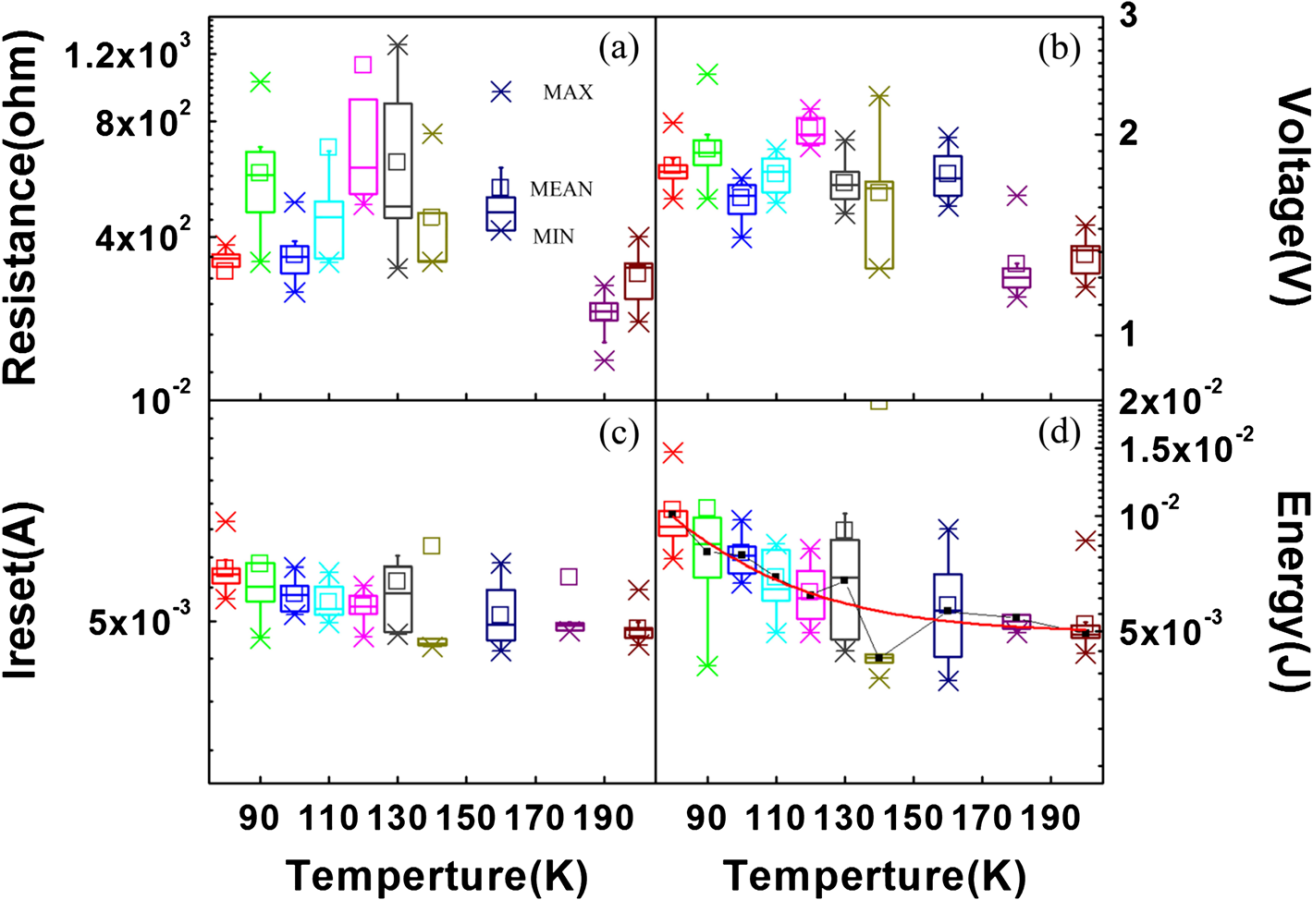
**Statistical distribution of device parameters and the calculated correlation between the energy versus sample temperature.** (**a**) LRS resistance (measured at 0.3 V), (**b**) RESET voltage, and (**c**) RESET current statistics at different temperatures. (**d**) Statistics on energy consumption during the RESET process as calculated. Here, the small square in the middle of the large square is the average mean value of the device parameters, and the large square indicates the distribution factors of 75% (top line) and 25% (bottom line), respectively. The black solid line in (**d**) is the average value line, and the red line is the statistical value fit line.

Figure 4 shows the statistical results of the typical electrical parameters of RRAM obtained at different temperatures. The LRS resistance, RESET voltage, and RESET current value distribution are shown in Figures 4a,b,c, respectively. The calculated integral energy consumption in the RESET at elevated temperature shows an exponential decrease in Figure 4d, as fitted by the solid line using the following equation:

(1) $$\begin{array}{ll} E_{\mathrm{cal}} & =\int_{0}^{t_{\mathrm{reset}}}\mathrm{VIdt}=\frac{I^{2}{}_{\mathrm{reset}}R^{2}}{3k}=E_{\exp}\\ & =5.49*\exp\left(197.8/T\right), \end{array}$$

where the voltage sweeping speed *k* = *V*/*t*, and *V*_reset_ is the reset voltage from LRS to HRS. Here, we suppose the identical energy dissipation of one cell in different RESET processes. The integration energy curve agrees well with the experimental fitting curve as shown in Figure 4d. The energy decays exponentially during the RESET with the elevated environmental temperature. Therefore, when charge detrapping dependence on environmental temperature is involved as in Equation 1, the calculated mean value of energy consumption in RESET decreased exponentially, which in good agreement with experimental results in Figure 4d. Although the switching parameters such as SET voltage, RESET current, and resistance of LRS or HRS vary with cycles, the statistical energy consumption still decays exponentially with the elevated environmental temperature when involving the charge trapping effect at low temperature.

**Figure 5:**
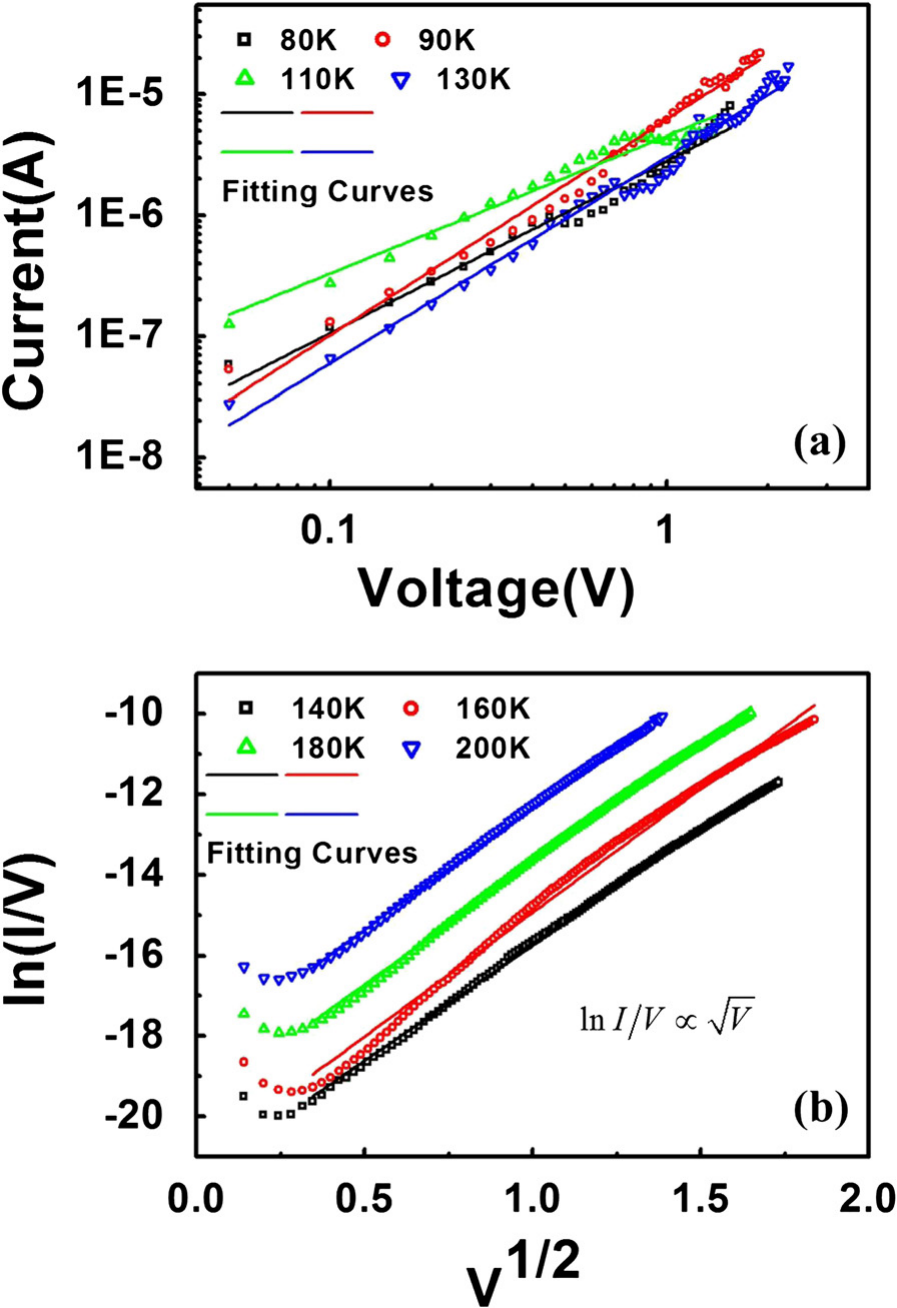
**Experimental *I*-*V *data of HRS at different temperatures.** (**a**) Linear fitting for the *I*-*V* curve at higher temperatures (80 to 130 K) using a log-log scale. The slope of the fitting curve is between 1 and 2, indicating that the conducting mechanism is electron hopping. (**b**) Experimental *I*-*V* data of HRS at higher temperatures (140 to 200 K). The good linear relationship between ln(*I*/*V*) and √*V* indicates that the electronic behavior of HRS can be predicted by utilizing Poole-Frenkel effect. *Y* coordinates of line were added with a constant to separate each line. The *V*^1/2^ in *x*-axis means √*V* in the (**b**), and it shows the good linear relationship between ln(*I*/*V*) and *V*^1/2^ in the temperature range 140 to 200 K obviously.

Figure 5 is the experimental *IV* data of HRS at different temperatures and the fitting curves by hopping and Frenkel-Poole conduction mechanism, respectively. The electron conduction in HRS of NbAlO at 80 to 130 K as shown in Figure 5a can be fitted well with hopping model because of the characteristic temperature dependence. A linear relationship between ln(*I*/*V*) vs. *V*^1/2^ can be obtained at 130 to 180 K as shown in Figure 5b. It indicates that the *IV* relation obeys the Frenkel-Poole conduction mechanism with the expression as in the equation below:

(2) $$I\propto V\exp\left(2\alpha \sqrt{V}/T-\mathrm{q\varphi b}/\mathrm{kT}\right),$$

where *I* is the current, *q* is the electron charge, *V* is the applied voltage, *α* is a constant, *b* is the energy barrier height, *k* is Boltzmann’s constant, and *T* is the temperature in Kelvin. Therefore, the transition temperature of 130 K from variable hopping conduction to Frenkel-Poole conduction for NbAlO HRS is confirmed and attracts research attention. It is believed that the density of trapped electrons or the local states in the oxide film play an important role as previous report described [15,16]. The temperature transition region should be different for different materials because of the local states and defect density differences.

## Conclusions

The conductive filament rupture in RRAM RESET process can be attributed not only to joule heat generated by internal current flow through a filament but also to the charge trap/detrapping effect. A new conduction mode is discussed from hopping conduction to Frenkel-Poole conduction with elevated temperature. This finding will help us understand the physical mechanism of resistive switching deeply in RRAM application.

## Competing interest

The authors declare that they have no competing interests.

## Authors’ contributions

PZ carried out the sample fabrication and drafted the manuscript. LY carried out the device measurements. QQS, PFW, AQJ, and SJD participated in the manuscript writing and discussion of results. DWZ participated in the design of the study and performed the statistical analysis. All authors read and approved the final manuscript.

## Authors’ information

PZ received his BS degree in Physics and his PhD degree in optics from Fudan University, Shanghai, China, in 2000 and 2005, respectively. He is currently an associate professor in the School of Microelectronics, Fudan University. His research interests include fabrication and characterization of advanced metal-oxide-semiconductor field-effect transistors, advanced memory devices, and graphene device. LY received his BS degree and the MS degree in microelectronics from Fudan University, Shanghai, China, in 2009 and 2012, respectively. He is currently a 28-nm Graphics Design Engineer in Huali Microelectronics Corporation, Shanghai. His research interests include low-power circuit, memory and device design, and fabrication for the cutting edge integrated circuit technology. QQS received his BS degree in Physics and his MS degree in microelectronics and solid state electronics from Fudan University, Shanghai, China, in 2004 and 2009, respectively. He is currently an associate professor in the School of Microelectronics, Fudan University. His research interests include fabrication and characterization of advanced metal-oxide-semiconductor field-effect transistors, mainly high-k dielectric-based devices. He is also interested in design, fabrication, and characterization of advanced memory devices, such as resistive switching memory devices and Flash. PFW received his BS and MS degrees from Fudan University, Shanghai, China, in 1998 and 2001, respectively, and his Ph.D. degree from the Technical University of Munich, München, Germany, in 2003. Until 2004, he was with the Memory Division of the Infineon Technologies in Germany on the development and the process integration of novel memory devices. Since 2009, he has been a professor ins Fudan University. His research interests include design and fabrication of semiconductor devices and development of semiconductor fabrication technologies such as high-k gate dielectrics and copper/low-k integration. AQJ is presently with a professor in the School of Microelectronics, Fudan University. He received his Ph.D. degree in 1999 in Studies of the Nanostructural Materials from the Institute of Solid State Physics, Chinese Academy of Sciences (Hefei). Later, he started his postdoctoral researches in the Institute of Physics (Beijing) (1999 to 2000) and Cambridge University (2001 to 2006). His main researches include nanotechnologies of nonvolative random access memories, such as ferroelectric memory (FeRAM), phase-change memory (PCRAM), resistor memory (RRAM), and Flash memory on the basis of CMOS, as well as the relevant device physics, especially about ferroelectric and semiconductor theories. SJD is a professor in the School of Microelectronics, Fudan University. He received his Ph.D. degree in Microelectronic and Solid State Electronics from Fudan University in July, 2001. From October 2001 to November 2002, he was a Research Fellow of Alexander von Humboldt Foundation with the Department of Materials Science and Engineering, Kiel University in Germany. From February 2003 to December 2004, he was a Research Fellow with the Silicon Nano Device Lab, National University of Singapore. DWZ received his BS, MSc, and Ph.D. degrees in Electrical Engineering from Xi’an Jiaotong University, Xi’an, China, in 1988, 1991, and 1995, respectively. In 1997, he was an associate professor in Fudan University, Shanghai, China, where he has been a full professor since 1999 and is currently the dean of the Department of Microelectronics and the director of the Fudan–Novellus Interconnect Research Center. He has authored more than 200 referred archival publications and is the holder of 15 patents. More than 50 students have received their MSc or Ph.D. degrees under his supervision. His research interests include integrated circuit processing and technology, such as copper interconnect technology, atomic layer deposition of high-k materials, semiconductor materials and thin-film technology; new structure dynamic random access memory (RAM), Flash memory, and resistive RAM; and metal-oxide-semiconductor FET based on nanowire and nanotube and tunneling FET.
